# Occupational Class Differences in Long-Term Sickness Absence Due to Breast Cancer during 2005–2013: A Population-Based Study among Finnish Women

**DOI:** 10.3390/ijerph16183477

**Published:** 2019-09-18

**Authors:** Johanna Suur-Uski, Johanna Pekkala, Jenni Blomgren, Olli Pietiläinen, Ossi Rahkonen, Minna Mänty

**Affiliations:** 1 Department of Public Health, University of Helsinki, FIN-00014 Helsinki, Finland; 2 The Social Insurance Institution of Finland, FIN-00100 Helsinki, Finland; 3 Department of Research, Development and InnovationLaurea University of Applied Sciences, City of Vantaa, FIN 01200 Vantaa, Finland

**Keywords:** sickness absence, breast cancer, occupational class differences, trend

## Abstract

Breast cancer is the most common cancer among women in Western countries with clear socioeconomic differences. Higher occupational class is associated with higher breast cancer incidence but with better survival from the disease, whereas lower occupational class is associated with higher risk of sickness absence. We are not aware of previous studies examining changes over time in occupational class differences in sickness absence due to breast cancer. This paper focuses on occupational class differences in the incidence and duration of sickness absence due to breast cancer over the period of 2005–2013. Age-adjusted occupational class differences in the cumulative incidence and duration of sickness absence due to breast cancer were calculated utilising a nationally representative 70% random sample of employed Finnish women aged 35–64 years (yearly N varying between 499,778 and 519,318). The results show that higher occupational class was associated with higher annual cumulative incidence of sickness absence due to breast cancer. Lower occupational class was associated with longer duration of absence. Occupational class differences in both cumulative incidence and duration of absence remained broadly stable. As a conclusion, these results suggest that measures should be targeted particularly to promotion of work capacity among employees with breast cancer in lower occupational classes.

## 1. Introduction

Abundant evidence indicates that occupational class, alongside with education and income, is among the most robust social determinants of health and disease [1,2]. Occupational class differences in health are widespread and persistent in Western countries with a hierarchical gradient: the lower the class, the poorer the health [1]. As for breast cancer, however, the pattern appears divergent. Higher occupational class is consistently associated with higher breast cancer incidence [3,4,5], although a more affluent occupational position tends to be related with better survival from the disease [4].

Breast cancer is the most common cancer among working-age women in both developed and developing countries [6], giving rise to notable costs [7]. There has been an increase in breast cancer incidence in Europe in the recent decades [8]. In Finland, breast cancer incidence is currently 93.95 per 100,000 individuals at risk [9], which is of a similar magnitude to other European countries [10]. As a result of effective treatments and preventive screenings, prognosis of breast cancer has improved, with increasing survival in many European countries [8]. In Finland, the 5-year relative survival rate has risen from 72% to 90% between the 1980s and 2010s [11].

Approximately half of the breast cancer cases are diagnosed in working-age women [12] and most women encountering it take sick leave during breast cancer treatment [13,14]. Sickness absence is an indicator of temporary inability to work due to ill health [15] and a well-established measure of health and well-being. The risk of sickness absence due to breast cancer has been associated with an advanced cancer stage [14,16], chemotherapy [13], old age [16], and also with lower occupational class [13,16]. The majority of women with breast cancer return to work within two years after the diagnosis [14,16] but low occupational class may negatively affect working capacity among employed women with breast cancer [13,16]. A U.S. study among employed women with breast cancer showed that manual work was associated with a lower likelihood of returning to work one year after breast cancer diagnosis [16]. Similarly, a Canadian study found that manual workers were more likely to be absent from work than workers in higher occupational classes during a three-year follow-up among female employees with breast cancer [13].

It is known from previous studies that occupational class is a major determinant of sickness absence [17] and that lower occupational class is associated with higher rates of all-cause sickness absence [18]. There is also ample evidence on occupational class differences in breast cancer morbidity [4]. According to our knowledge, studies on occupational class differences in breast-cancer-related sickness absence are scarce. Moreover, we are not aware of previous studies examining changes over time in occupational class differences in sickness absence due to breast cancer, despite the marked changes in breast cancer incidence and survival over the last decades. Therefore, the aim of this study was to examine occupational class differences in the incidence and duration of long-term sickness absence attributable to breast cancer among Finnish women from 2005 to 2013. Broad representative populations have not been utilised in the previous studies examining occupational class differences in breast-cancer-related sickness absence. Nation-wide evidence on the class differences could facilitate the identification of high-risk employee groups in terms of breast-cancer-related work disability, potential changes in these employee groups over time and, hence, targeting actions effectively at population level in the future.

## 2. Materials and Methods

This study is based on a nationally representative 70% random sample of employed Finnish women aged 35–64 years over the period 2004–2012, with a yearly number of women ranging between 499,778 and 519,318 followed from 2005 to 2013 for their sickness absences. The lower age limit was set at 35 years since breast cancer among young women has different clinical features and a poorer prognosis compared with older women [19]. The sample data was obtained from the register of the Social Insurance Institution of Finland (Kela). The format of the dataset is an unbalanced panel: individuals could be included in the sample each year between 2004 and 2012 or move in and out of the dataset (the inclusion was based on age, residence status in Finland and mortality). The sample data is representative of Finnish women aged 35–64 at the end of each year in 2004–2012.

### 2.1. Measurements of Sickness Absence

Data on sickness absence were based on the receipt of sickness allowance administered by and obtained from Kela. The register data on sickness allowance episodes from years 2005–2013 comprised the beginning and ending dates and diagnoses (based on the International Classification of Diseases, ICD-10) for each episode. For the purpose of this study, we selected sickness allowance episodes attributable to breast cancer on the basis of the ICD-10 code C50. Between 2005 and 2013, there were 20,389 sickness allowance episodes attributable to breast cancer in the data.

In the Finnish system, sickness allowance is granted to compensate for income losses due to work disability attributable to a disease up to approximately one year. A medical certificate is a prerequisite for the benefit. People with work disability aged 16–67 years can receive the benefit after a waiting period comprising the first day of disability and the following nine working days, i.e., calendar days excluding Sundays and midweek holidays. The definition of long-term sickness absence in this study is based on receipt of sickness allowance and, hence, refers to absence episodes lasting longer than 10 working days.

The outcomes of the study were the occurrence of new sickness absence episodes due to breast cancer during a given calendar year and the average number of sickness absence days in a sickness absence episode due to breast cancer each year over the period 2005–2013.

### 2.2. Measurements of Occupational Class

Year-end register data on occupational class were retrieved from Statistics Finland. These data were linked to the sickness allowance and sample data using the personal identity code assigned to each Finnish resident. Occupational classes were categorised according to the socio-economic classification of Statistics Finland, consisting of seven different categories [20]. The focus of this study was on employed women, and therefore, upper non-manual employees (yearly *N* between 111,127 and 128,905), lower non-manual employees (*N* between 270,426 and 287,016) and manual workers (*N* between 98,067 and 118,731) were included. We excluded old-age pensioners and disability pensioners because they are not eligible to receive sickness allowance. Entrepreneurs were not included due to their heterogeneous work status. Students, unemployed, and farmers were also not included in the analyses.

The study used solely secondary data retrieved from registers and therefore ethics approval was not required according to Finnish law [21]. Conventions of good scientific practice, data protection and information security have been applied in analysing the data and presenting the results.

### 2.3. Statistical Methods

The annual cumulative incidence of sickness absence due to breast cancer by occupational class was calculated from 2005 to 2013. Cumulative incidence is a person-based measure [22] and denotes the risk of having at least one new sickness absence episode during the study period [23]. The annual cumulative incidence in this study was expressed as per 100,000 individuals at risk with 95% confidence intervals (CI). When calculating the annual cumulative incidence, individuals with an ongoing sickness absence episode due to breast cancer from the previous year were excluded from the analyses in a given calendar year. For each study year, the population at risk for sickness absence was comprised of those women aged 35–64 years at the end of the previous year who did not have an ongoing absence episode due to breast cancer at the turn of the year in question.

Duration of absence due to breast cancer was calculated by occupational class for each calendar year during 2005–2013. Duration of absence is a time-based measure that denotes the average number of sickness absence days in a sickness absence episode [22,23,24]. Duration of absence was calculated annually by dividing the sum of sickness absence days in sickness absence episodes due to breast cancer by the sum of these episodes in each occupational class. Duration of absence is based on ended sick leave episodes [22], i.e., if the episode began during one year and ended during the next, all sickness absence days of that episode were included in the latter year. Duration of absence was presented with 95 per cent confidence intervals (CI).

For both measures, direct age-standardisation was carried out using five-year age groups, with the study population in 2008 as the standard population. Analyses were performed by using statistical programs SAS 9.4 (SAS institute, Inc. Cary, NC, USA) and R version 3.3.1. (R Core Team).

## 3. Results

Table 1 presents the occupational class distributions of the population at risk for sickness absence due to breast cancer for the years 2005, 2009 and 2013. Lower non-manual employees comprised the largest occupational class, being approximately twice as large as upper non-manual employees and manual workers. The proportions of both lower non-manuals and upper non-manuals increased and the proportion of manual workers declined from 2005 to 2013.

**Figure 1:**
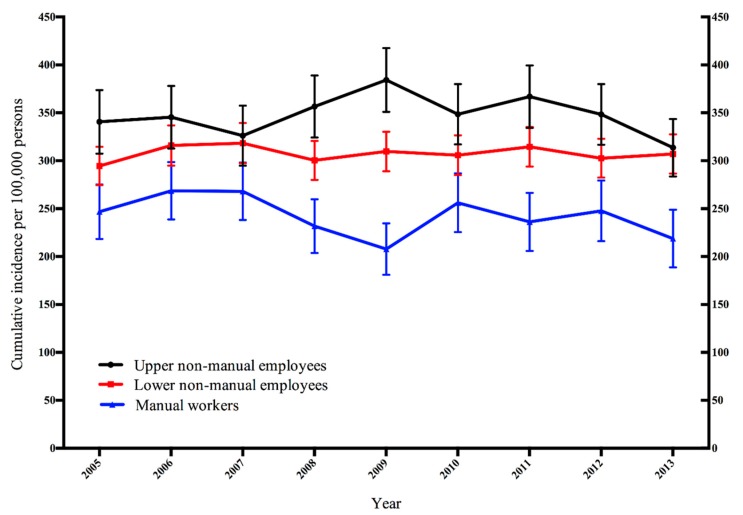
Age-adjusted annual cumulative incidence of sickness absence due to breast cancer by occupational class among Finnish women aged 35–64 years.

Figure 1 presents the age-adjusted annual cumulative incidence of sickness absence due to breast cancer. The cumulative incidence was highest among upper non-manual employees and lowest among manual workers with significant differences between those two study groups throughout the study period, except in year 2007 (Figure 1). The annual cumulative incidence ranged from 314 (95% CI 284–344) to 384 (95% CI 351–418) per 100,000 persons among upper non-manual employees and from 208 (95% CI 181–235) to 268 (95% CI 238–298) per 100,000 persons among manual workers. With regard to lower non-manuals, the age-adjusted annual cumulative incidence tended to be in between the latter two groups with the cumulative incidence ranging from 295 (95% CI 274–315) to 318 (95% CI 297–339) per 100,000 persons. Lower non-manual employees’ confidence intervals overlapped with either upper non-manuals or manual workers throughout the study with the exception of years 2008 and 2009. The cumulative incidence remained broadly stable in all occupational classes during the study period with the exception of 2009. In 2009, a slight increase took place among upper non-manual employees, whereas the cumulative incidence declined among manual workers. However, the occupational class differences in the cumulative incidence of sickness absence due to breast cancer remained broadly stable throughout the study period.

**Figure 2:**
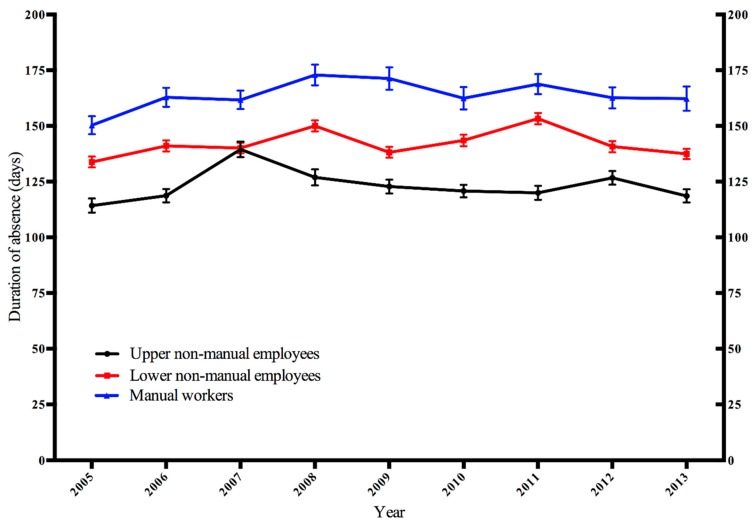
Age-adjusted annual duration of sickness absence due to breast cancer by occupational class among Finnish women aged 35–64 years.

Figure 2 presents the age-adjusted duration of sickness absence due to breast cancer by occupational class annually from 2005 to 2013. Manual workers had the longest duration of absence throughout the study period, ranging from 150 days (95% CI 149–152 days) to 173 days (95% CI 171–175 days). Upper non-manual employees’ duration of absence varied between 114 days (95% CI 113–116 days) and 140 days (95% CI 138–141 days). Duration of absence among lower non-manual employees was intermediate between upper non-manual employees and manual workers with the duration ranging from 134 days (95% CI 133–135 days) to 153 days (95% CI 152–154). Hierarchical occupational class differences in the duration of absence due to breast cancer remained significant throughout the study period with the exception of the year 2007, when the duration of absence was similar among upper and lower non-manual employees.

## 4. Discussion

This nationwide register-based study examined occupational class differences in the incidence and duration of long-term sickness absence due to breast cancer among employed women in Finland from 2005 to 2013. The main findings of the study are summarised as follows: (1) Higher occupational class was associated with higher cumulative incidence of sickness absence due to breast cancer across occupational classes throughout the study period. (2) An inverse occupational class gradient was found in the duration of absence: the lower the class, the longer the duration of absence due to breast cancer. (3) Occupational class differences in both cumulative incidence and duration of absence remained broadly stable during the study period.

This study found that women in higher occupational classes had higher sickness absence incidence due to breast cancer than those in lower occupational classes. The finding is contrary to a previous study [13] showing lower classes having more sickness absence than higher occupational classes in a small sample of Canadian women with breast cancer. However, the study was based on a sample of women already diagnosed with breast cancer. We were able to examine the association in a nationwide employed female population. The result in the present study is likely to reflect the well-established occupational class gradient in breast cancer incidence [4] but in addition the study brings novel insight to the development of occupational class differences in breast-cancer-related sickness absence over time.

Breast cancer is a severe disease, practically always compelling individuals to withdraw from work due to inability to perform job tasks as a result of the disease [9]. The risk factors for sickness absence are therefore likely to equal those that cause the disease, many of them varying by occupational class. The well-established risk factors for breast cancer comprise hormonal factors, including those relating to reproduction, such as low parity and high age at first birth [25]. In Finland, highly educated women have fewer children over their lifetime than less-educated women [26]. This phenomenon is likely to partly explain the results observed in this study. Furthermore, an increased risk of breast cancer has been previously shown to relate also to late menopause [25], which, in turn, is found to be associated with higher educational and occupational levels [27]. Other well-established risk factors for breast cancer comprise behavioural factors. Obesity, for example, is associated with an increased risk of breast cancer [28] and leisure-time physical activity with a decreased risk of both pre- and postmenopausal breast cancer [28]. Socioeconomic differences in health-related behaviours, however, show inverse socioeconomic gradients in developed countries. Obesity, for instance, is more common in lower classes, whereas engaging in physical activities at leisure time is more typical in higher classes [29].

A major part of the previously observed occupational class gradient in breast cancer incidence has remained unexplained even after adjustments for reproductive and lifestyle factors [3]. The aforementioned class differences in breast cancer risk factors may hence not fully explain the result of the present study regarding the cumulative incidence of sickness absence due to breast cancer. Previously, further explanations to the positive occupational class gradient in breast cancer risk have been suggested [30] to relate, for instance, to more stressful [31] and sedentary [5] work in higher occupational classes. On the other hand, women in higher occupational classes are more likely to attend mammography screenings compared to those in lower classes [32]. Mammography screening has been shown to increase breast cancer incidence but decrease mortality due to the disease [33]. This may also explain partly the observed positive association between occupational class and incidence of breast-cancer-related sickness absence in the present study. The class differences in the cumulative incidence remained broadly stable during the study period. In 2009 occupational class differences in the cumulative incidence widened temporarily as a result of a transient increase in the cumulative incidence among female upper non-manuals and a decline among manual workers. This coincided with an economic downturn and rising unemployment [34] in Finland. During the study period, there were no substantial changes in the sickness allowance system.

We found that lower occupational class was associated with higher average number of sickness absence days in a sickness absence episode due to breast cancer. The class differences in the duration of absence remained broadly stable over time. We are not aware of previous studies examining occupational class differences in duration of breast-cancer-related sickness absence. The difference in duration of absence between the highest and the lowest occupational class was at maximum 49 days in year 2011 and varied between 22–49 days during the study period. In other words, manual workers had sickness absence episodes that were generally around 30% longer compared to upper non-manual employees.

Duration of absence reflects the severity of sickness absence episodes [23] and could be used as an estimate of time spent in treatment or before returning to work [24]. Our finding is in line with a previous U.S. study showing that lower occupational class was associated with lower likelihood of returning to work 12 months after breast cancer diagnosis among employed women [16], and with a Canadian study with a corresponding outcome [13]. The result in the present study parallels the previous findings showing an inverse occupational class gradient in case fatality due to breast cancer, thus indicating better survival among women in higher occupational classes [4].

The class differences in breast cancer survival have been mainly explained by treatment factors, comorbidities, lifestyle factors, and stage at diagnosis which in turn is related to participation in mammography screenings [4]. These factors overlap largely with those causes previously found to associate with duration of sickness absence due to breast cancer. For example, receipt of adjuvant chemotherapy [35] and an advanced cancer stage [16,35,36] have been shown to associate with longer duration of absence and negatively affect the likelihood of returning to work. In a Swedish register study, the strongest association for sickness absence at 3 and 5 years after the diagnosis was with an advanced cancer stage [14]. Also, sickness absence days prior to the breast cancer diagnosis, considered an indication of comorbidities, have been shown to increase the risk of sickness absence three years after breast cancer diagnosis [14]. In Finland, women in lower income groups are diagnosed at a more severe stage of breast cancer compared to those in more affluent positions [12]. This holds true also among women aged 50–69 years who belong to the target group of mammography screenings [12]. Moreover, comorbidities tend to be more common among Finnish breast cancer patients from lower income groups [12].

Occupational class as a key indicator of socioeconomic position reflects, in addition to status and power, differences in work-related factors between employees across the hierarchy of occupational classes [37]. In all-cause sickness absence, various work factors, but in particular detrimental physical working conditions, have been major explanatory factors for the inverse occupational class gradient [17]. Working conditions may also play a key role in explaining the result regarding the duration of absence due to breast cancer in the present study. In manual occupations, work is more physically demanding; heavy lifting at work, for instance, has been shown to associate with lower likelihood of returning to work at one year after breast cancer diagnosis [16]. On the other hand, employees in higher occupational classes may have more flexibilities to adapt work tasks to better meet their current work ability. Breast cancer survivors in higher occupational classes are known to reduce their weekly workhours more [38,39]. Hence, one possible explanatory factor for our results might be that those in higher occupational class have the chance to work more flexible and shorter hours and therefore return to work more promptly. Accommodations at work and the employer’s encouraging attitude towards the facilitations have a major positive impact on return to work among female employees with breast cancer [16].

The present study has several strengths. To our knowledge, this is the first study to examine occupational class differences in sickness absence due to breast cancer in a nationwide employed female population over time. A vast nationally representative sample of working-age Finnish women was obtained from a comprehensive national register database and linked to register data on occupational classes and to data on sickness absence episodes of over 10 working days attributable to breast cancer over a nearly 10-year period. Sickness absence episodes were medically certified and based on paid sickness allowances, hence encompassing practically no missing information and self-report bias. Moreover, both person-based and time-based measurements of sickness absence were used in the study in order to give a profound insight into the health problem, as recommended previously [24]. With regard to the occupational classes incorporated into the study, the findings could be generalised to the Finnish female labour force and also with caution to other countries.

There are some limitations in the present study. In Finland, all sickness absence episodes of over 10 working days based on paid sickness allowances administered and registered by Kela can be tracked through the national registers, but no equivalent register database exists on shorter sickness absence episodes. We were therefore unable to examine the class differences in shorter breast-cancer-related sickness absence. However, this is not likely to cause serious bias since breast cancer tends to induce relatively long sickness absence episodes overall [36]. Lower non-manual employees’ cumulative incidence confidence intervals overlapped with either upper non-manual employees or manual workers during all other years except 2008 and 2009. However, occupational class gradient between upper non-manual employees and manual workers was found. Moreover, we were unable to examine the observed class differences in sickness absence attributable to breast cancer further due to lack of information on potential explanatory factors, such as reproductive history, menopause, health behaviours and working conditions, in the national database.

## 5. Conclusions

This nationwide register-based study among employed women found persistent occupational class differences in breast-cancer-related sickness absence over time. Lower occupational class was associated with lower cumulative incidence of sickness absence but longer duration of absence due to breast cancer. Manual workers consistently had longer duration of absence than employees in higher occupational classes which inevitably has economic effects on the employer, the society and the breast cancer patient. Critical appraisal of the reasons for the differences in duration of absence is warranted. Further research is needed on potential explanatory factors for the observed occupational class gradients in sickness absence due to breast cancer, such as differences in participation in mammography screenings, breast cancer treatment, health behaviours and working conditions. It is known that in Finland those in lower socioeconomic position are diagnosed at a more severe stage. Hence, employers might benefit from encouraging their employees to actively participate in mammography screenings and from more actively offering part time-employment for breast-cancer survivors in lower occupational classes. Additionally, national guidelines for administering leave of absence might help physicians in their daily work. In the future, measures should be targeted particularly to promotion of work capacity among employees with breast cancer in lower occupational classes.

## Figures and Tables

**Table 1:** Risk population and number of sickness absence (SA) episodes attributable to breast cancer by occupational class.

Occupational Class		Year		
		2005	2009	2013
Upper non-manual	Population at risk, *N* (%)	111,016 (22)	125,543 (24)	128,783 (25)
	Number of SA episodes	354	456	389
Lower non-manual	Population at risk, *N* (%)	270,150 (54)	279,000 (54)	286,411 (56)
	Number of SA episodes	766	869	894
Manual workers	Population at risk, *N* (%)	118,612 (24)	114,775 (22)	97,953 (19)
	Number of SA episodes	296	247	277
Total	Population at risk, *N* (%)	499,778 (100)	519,318 (100)	513,147 (100)
	Number of SA episodes	1416	1572	1560

## Abbreviations

CI	Confidence interval
ICD-10	The International Classification of Diseases, 10th revision
Kela	The Social Insurance Institution of Finland
WHO	World Health Organization
